# The factorial structure of the mini mental state examination (MMSE) in Japanese dementia patients

**DOI:** 10.1186/1471-2318-10-36

**Published:** 2010-06-09

**Authors:** Kenta Shigemori, Shohei Ohgi, Eriko Okuyama, Takaki Shimura, Eric Schneider

**Affiliations:** 1 Graduate School of Health Sciences, Seirei Christopher University, 3453 Mikatahara-Cho, Hamamatsu-City Shizuoka, 433-8558, Japan; 2 Hamamatsu Human Science Laboratory Ltd, 2254-10, Tomitsuka-Cho, Hamamatsu-City Shizuoka, 432-8002, Japan; 3 Research Laboratory on Bionics, Sanaruko Parktown 4-204 1864-1, Tomitsuka-Cho, Naka-ku, Hamamatsu-City Shizuoka, 432-8002, Japan; 4 Johns Hopkins School of Medicine, Center for Surgical Clinical Trials and Outcomes Research, 600 N. Wolfe St., Baltimore, MD 21205, USA

## Abstract

**Background:**

The Mini-Mental State Examination (MMSE) is one of the most commonly used instruments in the evaluation of global cognitive status. Few studies have investigated the relationship among its components in terms of factorial structure in Japanese individuals suffering from dementia. The aims of this study were: 1) to analyze the factorial structure of MMSE in Japanese dementia patients, 2) to clarify the MMSE static structure in identifying different cognitive profiles and understanding how these profiles are related to levels of dysfunction in subsets of dementia patients.

**Methods:**

30,895 consecutive outpatients with dementia were evaluated. The 11 subtests composing the MMSE and the global MMSE score were analyzed. Factor analysis based on principal component analysis with Promax rotation was applied to the data representing the frequency of failures in each subtest as identified by the MMSE.

**Results:**

Factor analysis identified three factors that explained approximately 44.57% of the total variance. The first factor, immediate memory, essentially constituted a simple index of the reading and writing subtests. The second factor, orientation and delayed recall, expressed the ability to handle new information. The third factor, working memory, was most closely related to the severity of dementia at the time of test administration.

**Conclusions:**

Japanese dementia patients appear to develop difficulty handling new information in the early stages of their disease. This finding, and our finding that there is a factor associated with disease severity, suggest that understanding the specific factors related to subtest items, which underlie the total MMSE score may be useful to clinicians in planning interventions for Japanese patients in the early stages of dementia.

## Background

The mini mental state examination (MMSE) is one of the most common tools to screen for cognitive impairment in older adults. The MMSE was developed to distinguish between older individuals with or without neuropsychiatric disorder early in the disease processes. It is also used during follow-up of patients suffering from cognitive impairment to assess disease progression. Folstein et al [1] reported that the MMSE is highly reliable on 24 hr (r = 0.89) and 28 day (r = 0.99) retest by single examiners. They also reported good inter-rater reliability for the MMSE (r = 0.83) when the MMSE was administered by two different examiners 24 hours apart. O'Connor et al [2] reported that 86% of respondents judged to have organic mental disorders scored 23 or less on the MMSE and that 92% of those judged to be cognitively intact scored 24 or more (sensitivity: r = 0.86, specificity: r = 0.92).

The MMSE asks questions that assess five areas of cognitive functioning (orientation, immediate memory, attention/concentration, delayed recall, language). Several studies have examined the component parts of the MMSE to investigate relationships among these components in terms of factorial structure. The first study to clarify MMSE factorial structure was by Fillenbaum et al [3]. These authors administered the MMSE to 36 patients with a diagnosis of probable Alzheimer's disease (AD) at its early stage. Factor analysis indicated that the composite score generated by multiple MMSE cognitive components could be explained by two factors, which together accounted for 66% of the variance. The first factor included attention/concentration, language and constructional praxis and the second comprised time-space orientation and delayed recall. In a longitudinal study, Tinklenberg et al [4] examined rates of change in score on each item in 63 probable AD patients. They discovered two significant factors. The first factor included naming, writing, immediate memory, reading a sentence and verbal comprehension. The second factor included constructional praxis, delayed recall, temporal orientation, attention/concentration and spatial orientation. They suggested that factor scores derived from the MMSE could be used to measure changes in the mental status of AD patients over time. Noale et al [5] analyzed data from 5,632 older adults, including individuals with dementia. They reported that the static structure of the MMSE was strongly influenced by each participant's potential to develop dementia. Different factorial structures were found for three different cognitive profile subgroups. Noale and colleagues suggest that analysis of MMSE static structure is useful to identify different cognitive profiles and understand the possible course of dementia in patients with cognitive impairment and AD.

For reasons related to culture and language, the factorial structure of the MMSE in dementia and/or AD patients might be different in Japan. However, to date, there have been no investigations of the factorial structure of the MMSE in Japan. The aims of this study are: 1) to analyze the factorial structure of MMSE in Japanese adults with dementia, 2) to clarify the MMSE static structure in identifying different cognitive profiles and understanding how these profiles are related to levels of dysfunction in subsets of dementia patients.

## Methods

### Patients (Table 1)

**Table 1:** Number (%) of subjects with correct answers on each MMSE subtest

MMSE Level	Numbers	temporal memory	spatial orientation	immediate orientation	attention/concentration	delayed recall	naming	verbal repetition	verbal comprehension	writing	reading a sentence	constructional praxis
Early (24-29)	10,861	7,760(71.4)	7,920(72.9)	10,689(98.4)	4,789(44.1)	2,678(24.7)	10,846(99.9)	10,785(99.3)	9,499(87.5)	10,844(99.8)	10,569(97.3)	9,465(87.1)
Moderate (15-23)	15,256	1,807(11.8)	2,440(16.0)	14,153(92.8)	988(6.5)	156(1.0)	15,118(99.1)	14,848(97.3)	10,042(65.8)	15,052(98.7)	13,296(87.2)	11,584(75.9)
Severe (0-14)	4,878	19(0.4)	14(0.3)	2,601(53.3)	8(0.2)	2(0)	3,820(78.3)	3,697(75.8)	708(14.5)	3,432(70.4)	1,695(34.7)	1,622(33.3)

*mean +/- SD: Early: 26.53 +/- 1.93, Moderate: 19.56 +/- 2.49, Severe: 9.90 +/- 3.38

This study examined older adults with a diagnosis of dementia presenting as outpatients between 1998 and 2005. The MMSE was administered to each of the patients by one doctor at their first diagnostic work-up and these scores form the basis of this analysis. And each patient received a comprehensive, multidisciplinary diagnostic evaluation. Diagnosis of dementia was based on the raw materials from the clinical interview, physical examination and neuropsychological tests. The interviewing doctor assessed health problems such as vascular risk factors (e.g. hypertension, heart disease, diabetes mellitus and hyperlipidemia), musculoskeletal problems (arthritis, fracture), gait disturbances, as well as hearing and visual deficits. The presence of previous or present major psychiatric disorders, serious neurological diseases, severe and uncontrolled arterial hypertension, diabetes mellitus, renal, hepatic or respiratory failure, anemia or malignancies were exclusion criteria. A total of 30,895 subjects, who fulfilled the abovementioned inclusion and exclusion criteria, were included in this study. During the study period, patient data accumulated in a patients' database system. Entry into this study was based on informed consent obtained from relative/proxy. The study was approved by the Ethics Committee of Seirei Christopher University (approval No. 09-033).

### MMSE

The MMSE was used to assess cognitive function in the study subjects. For the purpose of the present study, the 11 subtests composing the MMSE and the global MMSE score were considered independently. The MMSE is composed of 11 major items; temporal orientation (5 points), spatial orientation (5 points), immediate memory (3 points), attention/concentration (5 points), delayed recall (3 points), naming (2 points), verbal repetition (1 points), verbal comprehension (3 points), writing (1 points), reading a sentence (1 points), and constructional praxis (1 points). The MMSE has maximum score of 30, with five different domains of cognition analyzed: (1) Orientation, contributing a maximum of 10 points, (2) Memory, contributing a maximum of 6 points, (3) Attention and calculation, as a measure of working memory, contributing a maximum of 5 points, (4) Language, contributing a maximum of 8 points, and (5) Design copying, contributing a maximum of 1 point. Individuals scoring two points below the maximum in any independent domain (except design copying) were considered to be impaired. The MMSE was administered and scored by a medical doctor certified in internal-medicine with extensive dementia experience. (authors; M.K.).

### Data distribution

The distributions of individual partial scores were not normally distributed so, for the purpose of analysis, individual partial scores were converted to ranks ordered according to percentile parameters. The distributions in each subtest, as a function of the global MMSE score, were also evaluated for normality.

### Reliability

Cronbach's alpha is an index of reliability associated with the variation accounted for by the true score of the underlying construct where the construct is the hypothetical variable that is being measured. Reliability and internal consistency were evaluated by a correlation analysis followed by evaluation using Cronbach's alpha.

### Factor analysis

The 11 subtests composing the MMSE, as well as the global MMSE score, were analyzed. Factor analysis was applied to the data considering the frequency of failures in each subtest of the MMSE. Factor analysis was based on principal component analysis with promax rotation [6]. Partial scores (sub-totals) associated to each factor were computed for each subject and their distribution was examined as a function of the MMSE total score.

## Results

### Data distribution

Based upon MMSE total scores for the 30,895 individuals included in this study, dementia diagnoses were classified and distributed as follows: borderline dementia, 10,861 (MMSE score; 24-29); moderate dementia, 15,256 (MMSE score; 15-23); and severe dementia, 4,878 (MMSE score; 0-14). The mean MMSE score for the entire group was 20.48, (SD = 6.13) with the mean scores in the subgroups ranging as follows: Borderline: 26.53, (SD = 1.93), Moderate: 19.56, (SD = 2.49), Severe: 9.90, (SD = 3.38).

Table 1 shows the number (%) of subjects with correct answers on each MMSE each subtest. The borderline dementia group demonstrated problem in delayed recall and more than half of them could not succeed in tests of attention/concentration. Almost all participants in the moderate and severe dementia group showed disorientation in temporal and spatial orientation and demonstrated problems with calculation in serial 7 subtractions, which serves as a marker for problems with attention/concentration. In the moderate dementia group questions related to temporal orientation and spatial orientation were answered correctly at a lower rate. In the severe dementia group failure was most likely to be observed in, immediate memory, verbal comprehension, reading a sentence, and constructional praxis items. Naming, verbal repetition and writing items were not categorized for each group. This data would be related to characteristics of study population.

### Reliability

Table 2 shows the reliability analysis. Cronbach's coefficient exceeded 0.7 confirming the overall reliability of the MMSE when used to examine Japanese subjects. In Table 2, detailed analysis highlighted the lower homogeneity of some subtests. The subtests are ranked for decreasing correlation with the mean of the other subtests. The right column shows how Cronbach's alpha increases after the elimination of subtests with lower correlation scores. This supports the use of factor analysis to group homogeneous subtests.

**Table 2:** The reliability analysis

Subtests	correlation	cronbach alpha
1. temporal orientation	0.815	0.734
2. spatial	0.810	0.730
3. immediate memory	0.549	0.768
4. attention/concentration	0.772	0.752
5. delayed recall	0.571	0.765
6. naming	0.436	0.780
7. verbal repetition	0.403	0.783
8. verbal comprehension	0.647	0.756
9. Writing	0.488	0.780
10. reading a sentence	0.562	0.772
11. constructional praxis	0.420	0.777

### Factor analysis (Table 3)

**Table 3:** Factor analysis after Promax rotation

First factor	Second factor	Third factor	
**First factor: immediate memory**			
Naming	**0.79**	0.20	-0.17
verbal repetition	**0.68**	-0.18	-0.08
immediate memory	**0.66**	0.01	0.06
writing	**0.65**	-0.05	0.09
**Second factor: orientation and delayed recall**			
temporal orientation	-0.02	**0.80**	0.06
delayed recall	-0.06	**0.76**	-0.20
spatial	0.13	**0.68**	0.04
**Third factor: working memory**			
constructional praxis	-0.11	-0.13	**0.71**
reading a sentence	0.20	-0.05	**0.53**
attention/concentration	-0.09	0.32	**0.51**
verbal comprehension	0.36	0.03	**0.38**

Total	3.88	1.01	0.35
% of variance	35.23%	9.15%	3.19%
Cumulative%	35.23%	44.38%	44.57%

As shown in Table 3, factor analysis with promax rotation identified three factors that explained approximately 44.6% of the total variance. The first factor explained the 35.2% of the variance and included naming (factor loading; r = 0.79), verbal repetition (factor loading; r = 0.68), immediate memory (factor loading; r = 0.66) and writing (factor loading; r = 0.65). This factor essentially constituted a simple index of the reading and writing subtests.

The second factor explained 9.2% of the variance and included temporal orientation, delayed recall and spatial orientation. This factor included temporal orientation (factor loading; r = 0.80), delayed recall (factor loading; r = 0.76) and spatial orientation (factor loading; r = 0.68). The components of this factor are related to the processing of new information. Changes in these abilities are strongly associated with deteriorating cognitive function in the early stage of dementia.

The third factor explained the 3.19% of variance and included constructional praxis, reading a sentence and attention/concentration. This factor included constructional praxis (factor loading; r = 0.71), reading a sentence (factor loading; r = 0.53), attention/concentration (factor loading; r = 0.51) and verbal comprehension (factor loading; r = 0.38). This factor, which included items such as writing, reading, following commands and copying a design was most closely related to overall classification of dementia levels.

## Discussion

Among Japanese dementia patients, MMSE subtests are grouped into three main factors (immediate memory, orientation and delayed recall, working memory), which explain 44.6% if the variance. The main subtests loading on the first factor (35.2% of variance) are naming, verbal repetition, immediate memory and writing. Abilities assessed as part of the first factor were well maintained in individuals categorized with borderline or moderate dementia, as indicated by a total MMSE score of 15 or greater. MMSE subtests included the first factor evaluate verbal skills, reflect consolidated knowledge and refer to implicit memory. Previous studies have demonstrated that the cognitive abilities assessed by these subtests were well maintained in early dementia [7-9]. Also, the linguistic subtests of the MMSE were shown to be poorly sensitive to cognitive deterioration in a general dementia population [10,11]. Therefore, the abilities that make up the first factor appear to remain robust in Japanese dementia patients until they are severely affected by their disease.

The second factor explained 9.2% of the variance and included temporal orientation, delayed recall and spatial orientation. The second factor was highly sensitivity to cognitive impairment and included test items related to attention, delayed recall and spatial-temporal orientation. These items are known to be associated with episodic and semantic memory. Delayed recall mean measurements of new verbal and non-verbal material, temporal orientation describes the ability to pay attention to the date and to remember it, and spatial orientation are abilities related to topographical learning [12]. These items require both episodic and semantic memory and deficits in these areas are regarded as a hallmark of early AD. These difficulties are thought to arise due mainly to dysfunction in the hippocampus and mesial temporal lobe [13], and of their functional connection with the dorsolateral prefrontal cortex [14,15]. Therefore, difficulties with the subtest components that are a part this second factor, which is associated with both episodic and semantic memory, appear to be associated with the borderline (delayed recall item) and moderate (orientation item) stages in the progression of dementia. It is possible that careful consideration of the results of the subtests related to this factor can assist practitioners to better detect dementia in its earliest stages and better predict the probable course of the disease over time.

The third factor explained 3.2% of the variance and included constructional praxis, reading a sentence, attention/concentration and verbal comprehension. The third factor was most closely related to the earliest stages of dementia progress. These items are related to working memory abilities which is handling and learning new information [16]. According to Baddeley's neuropsychological model of working memory (WM) [17], WM is a short-term system that coordinates the temporary storage, simultaneous processing, and manipulation of information that is necessary in order to successfully perform complex cognitive functions and to learn new information. Constructional praxis seems to represent the visual component of WM, thus playing an important role in WM [18]. Attention/concentration expresses an indirect value of WM. Reading a sentence requires intact verbal comprehension. The impairment of verbal comprehension has been related to WM loss, rather than to impairment of linguistic knowledge, in dementia [19]. The third factor suggests that specific tests of WM may be very useful at the time of initial diagnosis of dementia.

As mentioned earlier, previous studies found two factors for AD in MMSE factorial analysis [3,4,20,21]. Fillenbaum et al [3] indicated two factors; first factor (praxis, language, attention/concentration and registration), second factor (delayed recall, temporal orientation and spatial orientation) in the MMSE to 36 patients with probable diagnosis of AD at early stage. Tinklenberg et al [4] also examined two factors; first factor (naming, writing, immediate memory, reading a sentence and verbal comprehension), second factor (constructional praxis, delayed recall, temporal orientation, attention/concentration and spatial orientation) in sample 63 probable AD patients. Brugnolo et al [21] also found two factors: their first factor was related to temporal orientation, delayed recall, attention/concentration, constructional praxis, spatial orientation and verbal comprehension and their second factor was related to reading a sentence, writing a sentence, naming, immediate memory and verbal repetition in a study of patients with mild to moderate AD.

However, in our analysis of MMSE performance in older adult dementia patients in Japan, three significant factors were identified. Our finding of three factors, rather than two as has been previously reported in patients with mild to moderate dementia, may be a function of differences in our study population. It is possible that patients we considered to have borderline dementia and included in our study might have been excluded from other studies. For example, Noale et al [5] reported finding three factors when the MMSE was used in a longitudinal cohort study that included healthy and dementia subjects. Also, Shyu et al [22] examined the factor structure and the contribution of underlying variables to the explanation of variability in MMSE scores of older adults in Taiwan and found that the same three structural factors explained much of the variance among the general elderly population, including those free of dementia. This suggests that MMSE static structures reflect the underlying cognitive profile of study subjects, as well as the progress and/or type of dementia when present.

There were several limitations of this study. First, this study was not limited to a single dementia type (e.g. AD) but instead it included individuals with all types of dementia, such as AD, Vascular Dementia, and individuals who might be classified as having mild cognitive impairment. This study was not able to distinguish the type of the dementia, because diagnostic methods have changed in Japan over the years between 1998 and 2005. The results of this study would have been more substantial if we had been able to include dementia type in our examination. For example, a diagnosis of AD implies acquired impairment in memory and other cognitive abilities that are sufficiently severe to interfere with daily functioning [23]. Executive dysfunction may be exhibited in the very stage of frontotemporal dementia, and the early symptoms of vascular dementia may be dependent on the locations of cerebrovascular disease lesions so having information on dementia type would have been useful in more clearly examining the factors that accounted for the variability in our sample. It is possible that having a mix of dementia types also contributed to our finding three factors where earlier studies of patients where dementia type was known found only two.

Another significant limitation of this study is that we used the MMSE to classify our subjects into the borderline, moderate and severe categories and at the same time, used the MMSE as our outcome of interest for the factor analysis. If it had been possible, it would have been better to classify disease severity using other methods which were unavailable to us, including the clinical dementia rating scale, or global deterioration scale.

## Conclusions

Factor analysis identified three factors which are related to the severity of dementia in older Japanese adults. The first factor represents a simple index including: naming, verbal repetition, immediate memory and writing. The second factor was related to memory: temporal orientation, delayed recall and spatial orientation. The third factor was related to complex tasks: constructional praxis, reading a sentence, attention/concentration and verbal comprehension. Lower MMSE subtest scores in each factor may provide clinician with an indication of the prognosis for dementia in a given patient. This study suggests that the MMSE static structure accurately reflects the cognitive profile of older Japanese adults. Further, this study suggests that the a clear understanding of the importance of individual MMSE subtest items could be useful to clinicians as they assess and plan interventions for patients with dementia in Japan.

## List of Abbreviations

MMSE: Mini Mental State Examination; AD: Alzheimer's disease; WM: working memory;

## Competing interests

Dr. Schneider has a pending patent on a treatment for acute neurologic injury. Otherwise, the authors declare that they have no competing interests.

## Authors' contributions

KS had role in developing the research question, data analysis and interpretation, and was the lead writer of this manuscript. SO had a major role in the conception and design of the study, supervised all aspects of the study's implementation, had a role in data analysis and interpretation, contributed to the writing of the manuscript, provided editorial comments. EO and TS contributed to the conception and study design, participated in data analysis and interpretation. ES contributed to the writing of the manuscript, and provided editorial comments. All authors read and approved the final manuscript.

## Pre-publication history

The pre-publication history for this paper can be accessed here:

http://www.biomedcentral.com/1471-2318/10/36/prepub
